# Addressing Mechanism of Fibrillization/Aggregation and Its Prevention in Presence of Osmolytes: Spectroscopic and Calorimetric Approach

**DOI:** 10.1371/journal.pone.0104600

**Published:** 2014-08-18

**Authors:** Sinjan Choudhary, Nand Kishore

**Affiliations:** 1 University of Mumbai & Department of Atomic Energy, Centre for Excellence in Basic Sciences, Santacruz (E), Mumbai, India; 2 Department of Chemistry, Indian Institute of Technology Bombay, Powai, Mumbai, India; National Institute for Medical Research, Medical Research Council, London, United Kingdom

## Abstract

Understanding the mechanism of protein fibrillization/aggregation and its prevention is the basis of development of therapeutic strategies for amyloidosis. An attempt has been made to understand the nature of interactions of osmolytes L-proline, 4-hydroxy-L-proline, sarcosine and trimethylamine N-oxide with the different stages of fibrillization of hen egg-white lysozyme by using a combination of isothermal titration calorimetry, differential scanning calorimetry, fluorescence spectroscopy, and transmission electron microscopy. Based on thioflavin T fluorescence emission intensities and microscopic images, the nucleation, elongation, and saturation phases of fibrillization have been identified. Isothermal titration calorimetry and differential scanning calorimetry have enabled a quantitative analysis of the nature of interactions of these osmolytes with various conformational states of lysozyme at different stages of fibrillization/aggregation. It is concluded that interaction of the osmolytes with lysozyme fibrils at both the nucleation and elongation stages are important steps in the prevention of fibrillization/aggregation. Identification of the nature of interactions is a key step towards the discovery and synthesis of target oriented potential inhibitors of these associations. This study is a first report in which calorimetry has been used to address interaction of potential inihibitiors with the protein at different stages of fibrillization.

## Introduction

The fibrillization or aggregation process in proteins involves several molecules forming higher order of conglomerates which have low solubility in aqueous medium. Depending upon their macroscopic morphology, such aggregates have been classified as ordered or disordered [1], [2]. Amorphous aggregates can be formed under physiological conditions at high concentrations in almost all proteins. However, β rich amyloid fibrils have been observed in a smaller set of proteins [2],[3]. The ordered aggregation in globular proteins occurs after partial unfolding of the native state into an intermediate state which is amyloidogenic in nature and exposes the aggregation prone regions [4]–[6].

The inability of a protein to adopt or remain in the native state can result into fibrillar and aggregated structures. This forms the basis of some of the most important neurodegenerative and metabolic disorders. Therefore, it is extremely important to discover the methods which lead to prevention of the formation of fibrillar/aggregated structures. This can be achieved by using suitable external agents which can act as potential inhibitors of these association processes. The small molecules which can alter the conformational stability or inhibit the protein fibrillization/aggregation have helped in the development of potential therapeutic strategies against the diseases which occur due to misfolding. Prevention of misfolding and aggregation of proteins by osmolytes has been reported in literature [7]–[11]. It is also known that in general the osmolytes enhance the thermal stability of a variety of proteins due to preferential hydration phenomenon [7], [8], [12]–[14]. The use of suitable small molecules permit a tremendous scope for their studies as potential therapeutic molecules against protein destabilization or several misfolding related disorders [15], [16].

Amongst the proteins which form amyloid fibrils under specific conditions, lysozyme is a suitable model to study the mechanism of amyloid formation and its prevention due to its small size and availability of extensive structural information in literature. For a long time lysozyme has been used as a model protein for understanding the complexity of protein structure and function in physiology and diseases [17], [18]. It has also been used as a model molecule for the investigation of enzyme catalysis, and as a disease marker [17].

Although the research over last few years has revealed the morphology and structural features of the amyloid/aggregated forms of the proteins, knowledge about the thermodynamics of amyloid formation and the process of inhibition is scarce. Evaluation of the thermodynamic parameters associated with interaction of potential inhibitors with proteins in the native, unfolded, and various stages of the fibrillization process can reveal the nature of interactions responsible for the inhibition process and hence identification of the functional groups on such molecules for effective inhibition.

In light of this background information, we have carried out calorimetric, spectroscopic and microscopic studies looking into the effect of osmolytes L-proline, 4-hydroxyl-L-proline, sarcosine, and trimethylamine N-oxide on lysozyme amyloid fibrillization. The main objective of this work is to apply quantitative techniques such as calorimetry in combination with spectroscopy and microscopy to unravel the energetics and mode of interaction of such small molecules with the protein which leads to prevention of fibrillization/aggregation. By combining the thermodynamic and structural details, it will be possible to understand the mechanism of interface in the fibrillization/aggregation process and hence suggest further guidelines towards the identification and synthesis of novel potential inhibitors.

## Materials and Methods

### Materials

Lysozyme (>0.95), L-proline (>0.99), 4-hydroxy-L-proline (>0.99), sarcosine (>0.98), trimethylamine N-oxide (>0.98) and thioflavin T (dye content: 0.65–0.75) were procured from Sigma-Aldrich Chemical Company USA. The listed purities of these compounds, on mass fraction basis, are given in the parenthesis. The solutions were prepared in deionized water which was double distilled and passed through Cole-Parmer mixed-bed ion exchange column. All the experiments were done in 40 mM phosphate buffer at pH 2.1 containing 100 mM NaCl. The stock solutions of lysozyme were dialyzed overnight at 4°C against the buffer with at least three changes of the latter. The osmolyte solutions were also prepared in the final dialysate buffer. The concentration of the protein and ThT were determined on a Jasco V-550 uv-visible double beam spectrophotometer, using a value of A_1 cm_^1%^ = 26.5 [19] at λ = 280 nm and *E* = 26,620 M^1^ cm^−1^ at λ = 412 nm [20], respectively.

### Fluorescence Spectroscopy

The steady state fluorescence measurements were done on a Cary Eclipse spectrofluorimeter with excitation and emission slit widths fixed at 2.5 nm. Thioflavin T (ThT), a cationic benzothiazole dye has been widely used to identify the amyloid fibrils having common structural features [21]–[24]. The ThT molecules were selectively excited at λ_ex_ = 450 nm [25]. The reported fluorescence emission spectra of the complexes have been corrected by subtracting the reference spectra of the control solutions containing same amount of the dye.

### Transmission Electron Microscopy

The visualization of the amyloid fibrils was done on a JEOL JEM-100B Transmission Electron Microscope which operates at an accelerating voltage of 80 kV. The Formvar-coated 300 mesh copper grids were used for deposition of the samples. The negative staining of the samples was done with 2% aqueous uranyl acetate solution. After pre-rinsing with large volumes of water, a 0.22 µm filter was used to filter the stains. Uranyl acetate is known to produce high electron density, image contrast, and impart fine grained impression to the image [26].

### Isothermal Titration Calorimetry

The interaction of amyloid fibrils with solvent and potential inhibitors was studied by using ultra sensitive isothermal titration calorimeters (VP ITC form Microcal LLC, and Nano ITC from TA Instruments). The fibril solution was titrated into the sample cell containing buffer or appropriate amount of the osmolyte in aliquots using a rotating stirrer-syringe of 250 µl capacity. The reference cell was filled with the respective buffer. The experiments were designed for a total of 25 consecutive injections, each having a volume of 10 µl of 0.606 mM native lysozyme solution or heat induced fibril solution into buffer or osmolyte solution in the cell. The duration between consecutive injections was 10 s with an interval of 4 min between each injection. The same procedure was used to measure the heats of dilutions by titrating buffer with the respective osmolytes at the same concentrations as used in the main experiments. After dilution corrections, the ITC profiles were analyzed to determine the heat of interaction by using Origin 7.0 and Nano Analyzer data analysis softwares supplied by Microcal and TA Instruments, respectively.

### Differential Scanning Calorimetry

A Nano DSC from TA Instruments, having capillary cells of volume 300 µl was employed to study the thermal transitions of the protein under different conditions. The sample cell of the DSC contained protein or protein+osmolyte solution and the reference cell was filled with the corresponding buffer or buffer+osmolyte solution. All the DSC experiments were done at a scan rate of 1 K min^−1^. The reported excess heat capacity *versus* temperature scans for the protein transitions have been corrected for the corresponding solvent *versus* solvent scans heated under the same conditions.

## Results and Discussion

### Binding of Thioflavin T with amyloid fibrils

An indepth knowledge of the binding mode of ThT with amyloid fibrils is essential for understanding the structural characteristics of these associated assemblies. An important advantage of ThT compared to other amyloid specific dyes such as Congo Red is its ability to detect the amyloid fibril formation *in situ* [27]. The main aim of the present study is to understand the energetics and mechanism of interaction of some potential inhibitors of aggregation/fibrillization with the protein in the native and at different stages of fibrillization. For this the changes in fluorescence properties of ThT have been explored upon interaction with these states of the protein.

**Figure 1:**
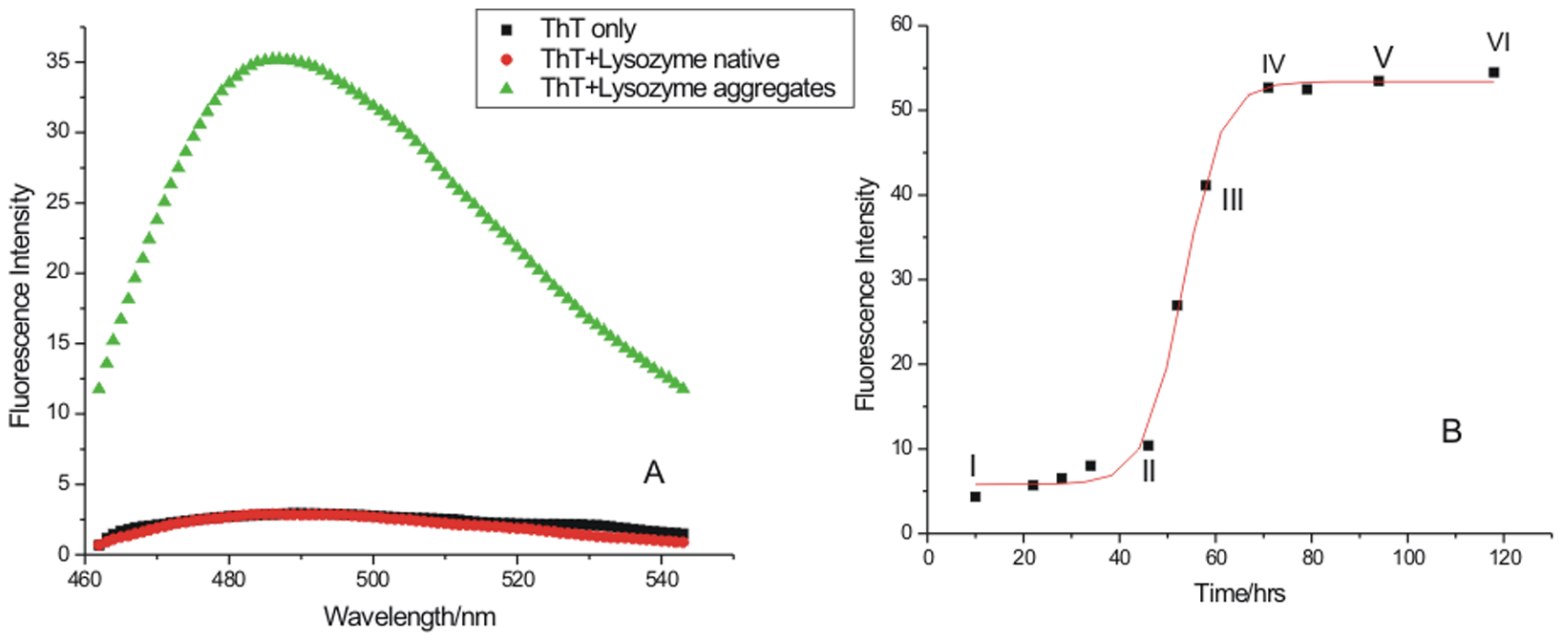
Fluorescence emission intensity accompanying the binding of ThT with amyloid fibrils after 72 h of incubation in the presence of (▪) ThT, (•) ThT + native lysozyme, (▴) ThT + lysozyme aggregates (Panel A); and kinetics of the lysozyme amyloid formation (Panel B), point I shows lysozyme at the native state, point II denotes the end of nucleation period and beginning of the elongation stage, point III shows lysozyme at the middle of the elongation period and points IV, V and VI denotes the saturation stages of lysozyme.

Figure 1A shows the fluorescence emission spectra accompanying the binding of ThT with lysozyme amyloid fibrils. ThT selectively interacts with amyloid fibrils which leads to significant changes in its fluorescence emission at λ_em_ = 480 nm when excited at λ_ex_ = 450 nm [28]. No fluorescence emission was seen with ThT in buffer and ThT in presence of native lysozyme at 25°C (Figure 1A). Significant fluorescence emission was seen when ThT was interacted with the heat induced fibrils which were obtained after 72 h of incubation at 57°C (Figure 1A). The use of ThT as a fibril specific dye permitted monitoring the process of mature fibril formation as a function of time. For this a solution of native lysozyme (690 µM), prepared in 40 mM phosphate buffer containing 0.1 M NaCl at pH 2.1 was incubated at 57°C. A calculated amount of lysozyme solution was taken from this solution and added to ThT solution containing the same buffer, such that the final concentrations of the protein and the dye in the solution were 69 µM and 50 µM, respectively. NaCl was used as it is known to promote fibril extension [29]–[32]. A typical sigmoidal curve was obtained which has the characteristics of fibril extension (Figure 1B). The curve contains three distinct phases: nucleation (region I–II), fibril extension (region II–IV) and saturation (region IV–VI). These three stages contain varying degree of fibrillization in lysozyme (Figure 1B). As seen in this figure, the fluorescence emission intensity of ThT remains nearly the same from state I (incubation time 10 h) to state II (incubation time 48 h) where the nucleation phase ends and the elongation phase begins sharply. It is seen that the elongation of fibrils rises sharply from 48 h to 61 h of incubation (stage III). After 70 h of incubation, the extent of fibril formation appears to be saturated (region IV to VI). The structural features of the native protein and at these three phases were characterized by TEM. For thermodynamic characterization of different fibrillar states of lysozyme in the absence and presence of different osmolytes, isothermal titration calorimetric studies were performed.

### Structural characterization of fibrillar extension by transmission electron microscopy

**Figure 2:**
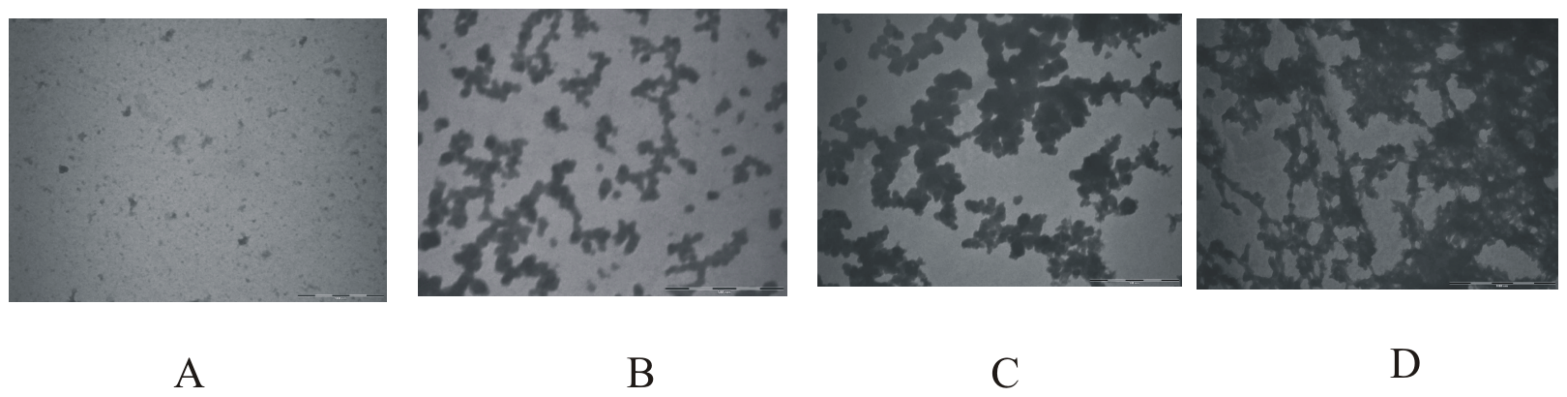
Transmission Electron Microscopic images of lysozyme solution (A) in the native state, and after the incubation at 57°C for (B) after 48 h, (C) 61 h and (D) 90 h which correspond to the stages I, II, III and V of Figure 2B, respectively.

To characterize different structures formed at various stages of fibrillization, Transmission Electron Microscopy (TEM) was used. The TEM image of native lysozyme at pH 2.1 is shown in Figure 2A. The fibrillar extension in lysozyme solution after incubation at 57°C for 48 h, 60 h and at 90 h are shown in Figure 2B to 2D. Figure 2 shows that at 57°C the morphology of the protein changes from non-aggregated state (Figure 2A) to a fibril network as the time proceeds. After 48 h it is seen that the oligomeric structures of lysozyme are formed which provide nucleus for further extension, as is observed in Figure 2C which corresponds to incubation for 60 h. After 90 h, a clear mature fibrillar structure is observed as shown in Figure 2D.

### Isothermal titration calorimetry of dilution of lysozyme in the native state and heat induced lysozyme fibrils in buffer

**Figure 3:**
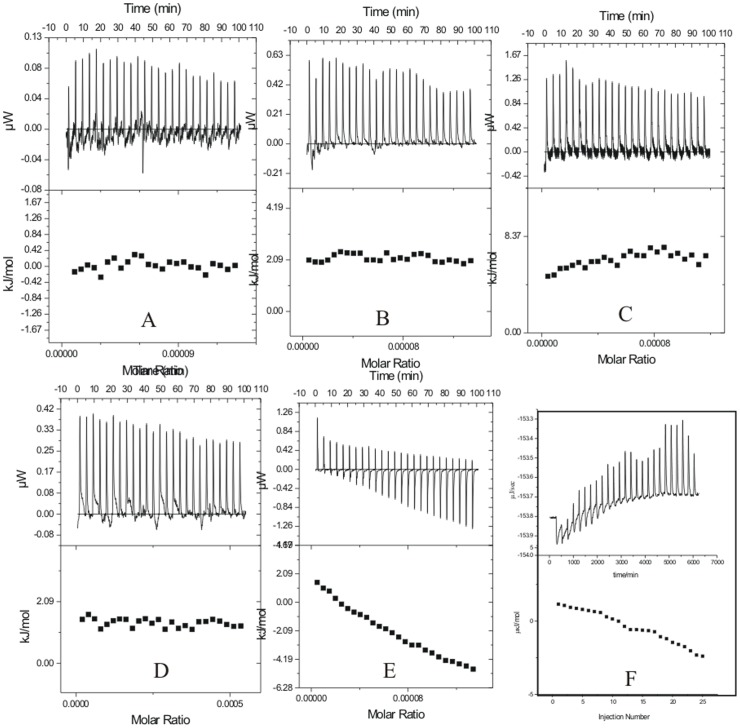
Representative ITC profiles for the titration of (A) lysozyme with buffer at 25°C, and (B) lysozyme at 37°C (C) lysozyme at 60°C, and heat induced aggregates of lysozyme after incubation period of (D) 48 h, (E) 61 h, and (F) 90 h.

There are no reports in literature on the energetics of interaction of inhibitors with proteins at different stages of the aggregation/fibrillization process. Experiments were designed on interaction of the fibrillar protein with different osmolytes. However, prior to that the thermodynamic characterization of the dilution of native lysozyme and at three stages of the fibrillization process in aqueous medium were done by using isothermal titration calorimetry. The protein solution was taken in the syringe of ITC whereas both the reference and sample cells were filled with the buffer. Experiments were conducted at different time intervals of fibrillization and the respective representative ITC profiles are shown in Figure 3. The ITC results suggest that the dilutions of lysozyme into buffer from the native state, denatured state, or aggregated/fibrillar states do not show appreciable heat effects.

### Fluorescence kinetic studies of the formation of lysozyme aggregates in presrence of different osmolytes

**Figure 4:**
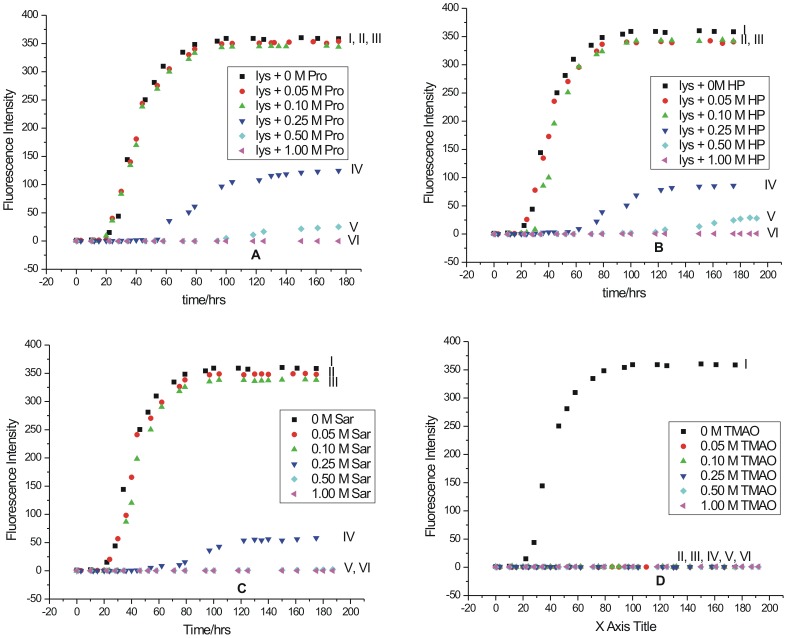
Kinetics of lysozyme fibril extension in absence and in presence of different concentration of osmolytes (A) L-proline, (B) 4-hydroxy-L-proline, (C) sarcosine, and (D) trimethylamine-N-oxide studied by monitoring the changes in fluorescence emission intensity as a function of time.

Figure 4 shows a typical sigmoidal path during the fibrillization process having three stages: nucleation, elongation and saturation. The kinetics of the fibrillization process was studied by using 690 µM lysozyme in presence of different osmolytes. The process of fibrillization process was monitored by using 50 µM ThT. The osmolytes chosen for these studies were L-proline, 4-hydroy-L-proline, sarcosine and trimethylamine N-oxide (TMAO). The time course of the fibril extension was examined on fluorescence spectrophotometer at 57°C. Here also a calculated amount of lysozyme solution was taken and added to the ThT solution in the absence and presence of different osmolytes, such that the final concentrations of the protein and the dye in the solution were 69 µM and 50 µM, respectively. The length of the nucleation and elongtation phases under each of these conditions are reproducible within an error of 10%. In Figure 4, the curves represent formation or prevention of fibrils formation in the absence (I) or presence of 0.05 M (II), 0.10 M (III), 0.25 M (IV), 0.50 M (V), and 1.00 M (VI) osmolytes.

Figure 4A shows the progress of the fibril formation as a function of time monitored by the ThT binding assay in the absence and presence of different concentrations of L-proline. The curve I in this Figure represents formation of fibrils in lysozyme in the absence of any additive. In the presence of 0.50 M L-proline (curve V), the duration of the nucleation phase increased substantially as reflected by significant reduction in the fluorescence emission intensity of ThT. This suggests that L-proline at this concentration has acted as an inhibitor of the lysozyme fibrillization process. When the concentration of L-proline was increased to 1.0 M in solution, no binding of ThT with lysozyme was observed as reflected by absence of the fluorescence emission under these conditions (Curve VI). These results suggest that 1.0 M L-proline is able to arrest the fibrillization process in lysozyme yielding a very clear solution. Figure 4B represents the effect of 4-hydroxy-L-proline on fibrillization of lysozyme as a function of concentration and time. It is seen that 0.50 M 4-hydroxy-L-proline is able to delay the nucleation and elongation periods much more than L-proline. It is further observed that the fluorescence emission intensity of ThT also decreased many folds in presence of 0.50 M 4-hydroxy-L-proline compared to that of L-proline. This suggests that 4-hydroxy-L-proline is a stronger inhibitor of fibrillization than L-proline which differs by one –OH group in its chemical structure. Here also 1 M 4-hydroxy-L-proline is able to suppress the fibrillization completely (curve VI). Figure 4C shows the effect of sarcosine on fibrillization of lysozyme as a function of concentration and time. Similar to the effect of 4-hydroxy-L-proline, sarcosine at 0.50 M concentration level is also able to delay the nucleation and elongation periods significantly. Here again 1.0 M sarcosine is able to suppress the fibrillization completely. Figures 4A to 4C demonstrate that up to a concentration of 0.10 M, proline, 4-hydroxy-L-proline or sarcosine are not effective in the prevention of formation of fibrils. These osmolytes exhibit significant effect at a concentration of 0.25 M and above.

The effect of TMAO on fibrillization process is shown in Figure 4D. It is seen that at all the studied concentrations of TMAO, the fluorescence intensity of ThT totally vanished. The fibrils of lysozyme after an incubation period of 80 h were clearly visible with naked eye. However, in the presence of L-proline, 4-hydroxy-L-proline and sarcosine, the solution after the same incubation period was clear. In the case of TMAO, the solution after an incubation period of 80 h contained aggregates which were observed to be amorphous in nature. It is known that ThT does not bind to amorphous aggregates but binds specifically to the fibrillar structures [33], [34]. Thus TMAO is observed to follow a different mode of action compared to other osmolytes. It either has a little effect [35] or may induce oligomerization [36] in case of Aβ40 proteins. It can induce formation of folded oligomers or enhance fibrillation [37] in α-synuclein. It can also induce formation of amorphous aggregates in polyQ peptides [38].

### Transmission Electron Microscopy

**Figure 5:**
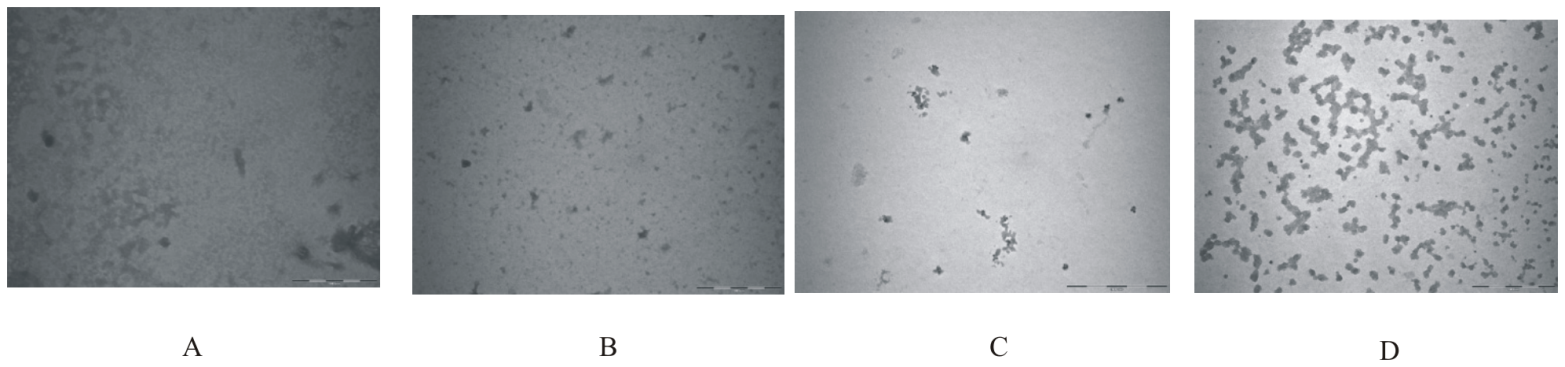
TEM images of lysozyme under different incubation conditions: images (A), (B), (C) and (D) are those for lysoyme solution incubated at 57°C in presence of L-proline, 4-hydroxy L-proline, sarcosine and TMAO respectively.

Figure 5 shows the TEM images of lysozyme after it was incubated at 57°C for a period of 72 h in presence of 1 M L-proline, 4-hydroxy-L-proline, sarcosine and TMAO. It is clear that no fibrils are present under these conditions in the presence of 1 M L-proline, 4-hydroxy-L-proline and sarcosine (Figure 5A, 5B and 5C). However, when the solution of lysozyme was incubated at 57°C for 72 h in presence of TMAO small aggregates were observed which do not resemble the fibrillar structure (Figure 5D). Thus it is clear from these results that aggregation/fibrillization can be blocked effectively if the osmolytes are present in solution before the aggregation begins.

### Calorimetric studies on the interaction of osmolytes with lysozyme fibrils

The thermodynamic characterization of the mechanism of inhibition of fibrillization by osmolytes was done by ultrasensitve isothermal titration calorimetry. The experiments were performed at different concentrations of osmolytes L-proline, 4-hydroxy L-proline, sarcosine and TMAO. Solutions of lysozyme at different phases of the fibrillization process were titrated with the osmolytes to understand the energetics and nature of interactions operating in these systems. We could not find any studies in literature in which the interaction of potential inhibitors with the protein at different stages of the fibrillization process has been addressed specifically based on heats of interaction. It is understood that since the protein may not have specific binding sites for the inhibitors, the challenge lies in identifying the nature of interactions and hence the properties of the molecules responsible for the inhibition process.

### Isothermal titration calorimetry of lysozyme fibrils at the nucleation phase

**Figure 6:**
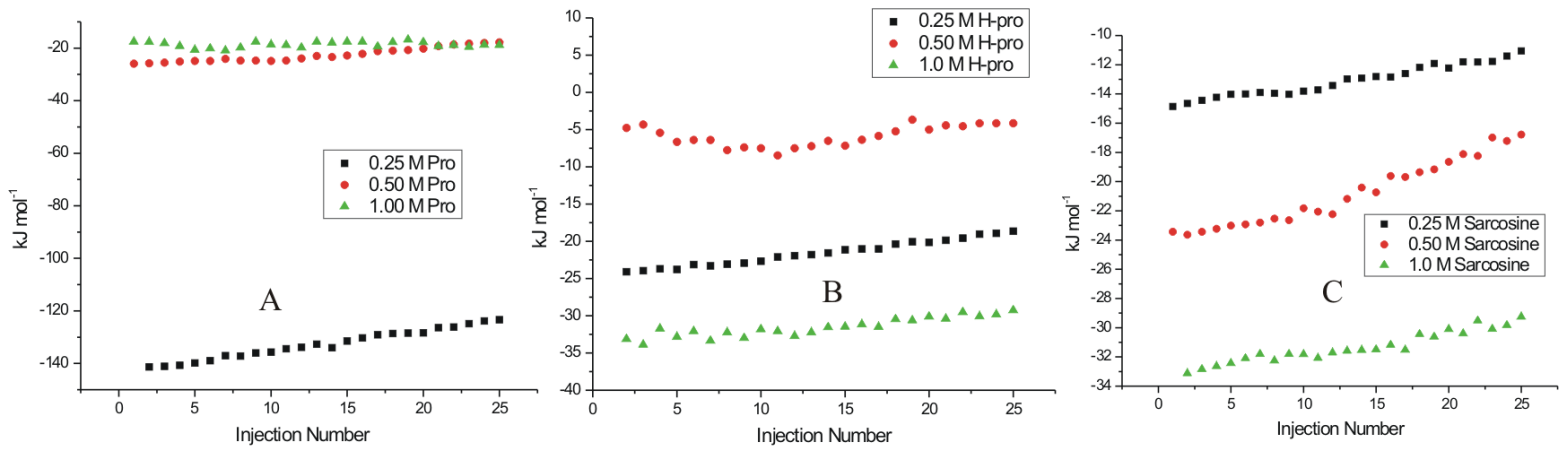
ITC profiles for the titrations of lysozyme at nucleation stage (after 48 h of incubation) with (A) L-proline (B) 4-hydroxy-L-proline and (C) sarcosine at 25°C.

Figure 6 represents the heat evolved when lysozyme, at the nucleation phase of fibrillization after 48 h of the incubation period at 57°C, is interacted with L-proline. The solution of lysozyme chosen for the ITC studies under these conditions corresponds to point II of Figure 1B. In the ITC experiments, different concentrations of osmolytes were taken in the sample cell, and lysozyme solution was taken in the syringe. It is seen in Figure 6A that interaction of L-proline with lysozyme at the nucleation phase is exothermic in nature. The extent of exothermicity decreases with increase in the molar ratio of lysozyme to L-proline. It is known that the propagation of aggregation begins from partially folded or unfolded species that expose a greater backbone surface area than the native protein. According to Auton and Bolen [39], the unfavorable interactions of L-proline with the peptide backbone cause preferential exclusion of the solute from the protein–water interface of these partially folded intermediates, hence increasing its free energy. It is known that the structural stability of proteins can be enhanced by certain osmolytes through the enhancement of hydrogen bonding in the hydration shell of the macromolecule [40]. This whole mechanism provides exothermic contribution to the overall reaction which is reflected in the ITC measurements. The enthalpy of interaction of lysozyme fibrils with L-proline at nucleation stage is most exothermic with the values changing from −(141.2±3.2) kJ mol^−1^ to −(123.5±2.5) kJ mol^−1^ of the protein when the concentration of osmolyte is 0.25 M. The extent of exothermicity decreased at higher concentration of the osmolyte with the values varying from −(26.6±2.4) kJ mol^−1^ to −(18.5±2.3) kJ mol^−1^ of the protein with 0.5 M L-proline and even further lower [an average of −(18.6±2.7) kJ mol^−1^] with 1 M L-proline. At higher concentration, L-proline is known to form supramolecular structures [41]. Therefore lesser availability of the polar groups of L-proline as a result of supramolecular structure results in reduction of polar interactions between L-proline and protein, and hence decreased exothermicity. It has also been reported [41] that L-proline having amphiphilic nature of its supramolecular assembly, provides hydrophobic surfaces which interact with aggregation prone solvent exposed hydrophobic residues during folding of the protein thereby blocking the protein aggregation effectively. The interaction of lysozyme at the nucleation stage with 4-hydroxyl-L-proline and sarcosine are also observed to be exothermic in nature (Figure 6B). A comparison of the enthalpy of interaction of lysozyme with L-proline and 4-hydroxy-L-proline suggests that additional exothermicity observed with the latter is due to an extra hydroxyl group present in it which can interact with the polar groups of the protein via hydrogen bonding. Similar effect is also observed in the interaction of lysozyme at the nucleation phase with sarcosine (Figure 6C).

### Isothermal titration calorimetry of interaction of lysozyme fibrils at the elongation phase with different osmolytes

**Figure 7:**
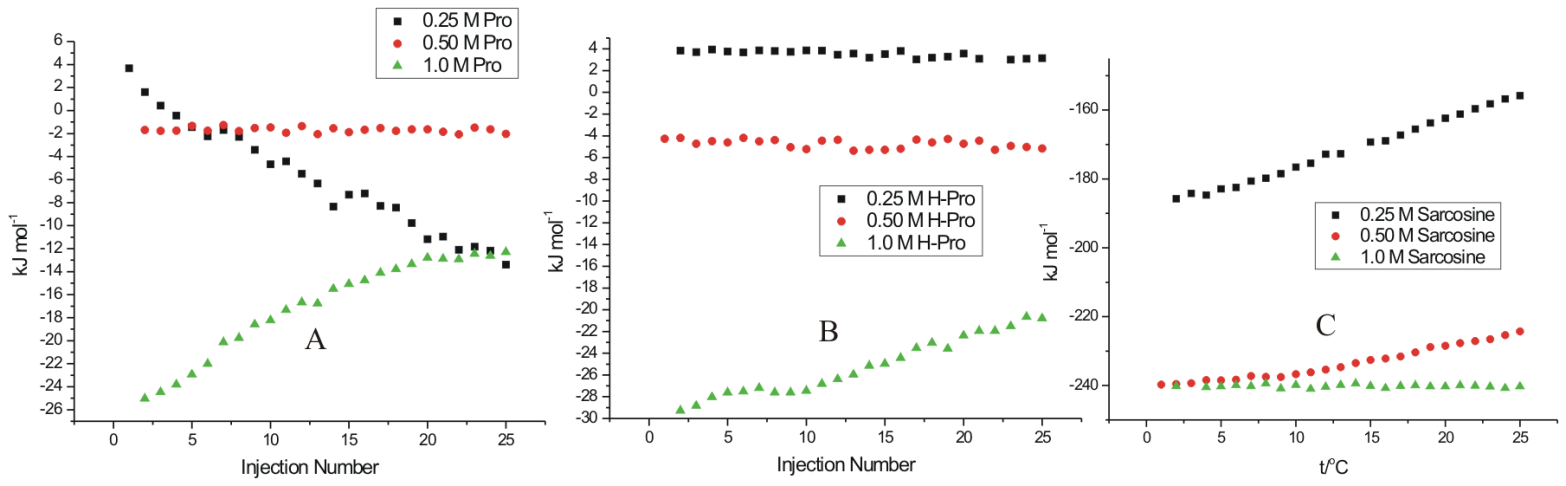
ITC profiles for the titrations of lysozyme solution at elongation stage (after 61 h of incubation) with (A) L-proline (B) 4-hydroxy-L-proline and (C) sarcosine at 25°C.

Figure 7 represents the heat profiles accompanying the titration of lysozyme fibrils with osmolytes at the elongation phase corresponding to point III in Figure 1B. It is seen in Figure 7 that the titrations of the fibrils with L-proline, 4-hydroxyl-L-proline and sarcosine are mostly exothermic in nature, highest with sarcosine. It is observed that under these conditions, L-proline does not show appreciable heat of interaction or a specific heat pattern (Figure 7A). The maximum value of heat of interaction of lysozyme fibrils with L-proline under these conditions is -(25.6±3.3) kJ mol^−1^ of the protein. The protein species at the nucleation stage will have greater exposed backbone surface area than the native state and are thus preferentially stabilized by L-proline. But with the late aggregates having high molecularity and a significantly smaller exposed surface, the favorable osmolyte interactions would be relatively diminished [42], which is reflected in the less exothermic contribution of the interaction of L-proline with fibrils at the elongated stage.

Similarly for 4-hydroxy-L-proline, the heat of interaction is very small (Figure 7B). For example the enthalpy of interaction of lysozyme at this stage with 0.25 M 4-hydroxy-L-proline is in the range of 3.8±0.3 to 3.1±0.3 kJ mol^−1^ which reaches a maximum range of [−(29.3±2.2) to −(20.8±2.6) kJ mol^−1^) for interaction with 1 M 4-hydroxy-L-proline (Figure 7B). Interestingly 0.25 M sarcosine and 0.5 M sarcosine show significant heat of interaction with the fibrils at the elongation phase (Figure 7C). The isothermal titration calorimetry resuls on the interaction of lysozyme in the native, nucleation stage and elongation stage with the osmolytes L-proline, 4-hydroxy-L-proline, and sarcosine suggest that these osmolytes preferentially interact and possibly inhibit the aggregation/fibrillization at both the nucleation stage and elongation stage where the heat of interaction is observed to be higher than that with the native state, and denatured state of the protein.

### Interaction of matured fibrils with L-proline

**Figure 8:**
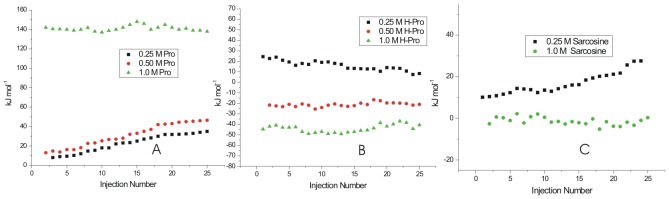
ITC profiles for the titrations of lysozyme solution at saturation stage (after 90 h of incubation) with (A) L-proline (B) 4-hydroxy-L-proline and (C) sarcosine at 25°C.

Figure 8 represents the dilution corrected integrated heat profile for the titration of matured lysozyme fibrils with the osmolytes L-proline, 4-hydroxyl-L-proline and sarcosine. For interaction with 0.25 M L-proline it is observed that as the number of injections (or the concentration of fibrils) increases in the sample cell, the trend of the heat change moves towards more endothermicity from a value of 8.2±1.2 to 35.4±2.2 kJ mol^−1^ (Figure 8A). Similar behavior is seen when the matured fibrils are titrated into 0.50 M L-proline solution (Figure 8A) where again the heat change becomes more endothermic as more fibrils are added to the osmolyte solution. Although, as seen in this Figure, there is no general trend in the heat change observed. The interaction of the matured fibrils with 1 M L-proline is endothermic with an average value of about 145±3 kJ mol^−1^ of the protein. In general it is seen that the interaction of the matured fibrils with L-proline is endothermic in nature.

### Interaction of matured with 4-hydroxy-L-proline fibrils

4-hydroxy-L-proline and L-proline differ by a hydroxyl group. The effect of interaction of this additional hydroxyl group is shown in the Figure 8B which shows the dilution corrected integrated heat profile for the titration of matured fibrils with different concentrations of 4-hydroxy-L-proline. Unlike in the case of L-proline, the enthalpy of interaction changes from endothermic [(25.1±1.4) kJ mol^−1^ of protein] towards lesser endothermic [(10.2±1.8) kJ mol^−1^] values as the number of injections increases in the solution. When the titrations are carried out in 0.50 M 4-hydroxy-L-proline, there is no definite trend in the heat of interaction observed at higher concentration of the fibrils (Figure 8B). The heat of interaction is exothermic with an average value of −(22.4±1.9) kJ mol^−1^. With 1 M 4-hydroxy-L-proline in the sample cell, the heat of interaction is further more exothermic with an average value of −(50.3±2.3) kJ mol^−1^. In general, the heat of interaction of the matured fibrils is exothermic with 4-hydroxy-L-proline due to additional –OH group present in it compared to L-proline.

### Interaction of matured fibrils with sarcosine

Figure 8C shows the integrated heat profile for the interaction of matured fibrils with sarcosine. Here a small trend in the heat of interaction is observed as the concentration of the fibrils increases in 0.25 M sarcosine in solution taken in the cell. This trend is more towards the endothermic nature. However, when the concentration of sarcosine is increased to 1 M in the solution, the heat of interaction of the fibrils do not show a definite trend as the number of injections of the matured fibrils in the sample cell is increased and the overall heat change is very small.

### Interaction of different stage of fibrils with TMAO

**Figure 9:**
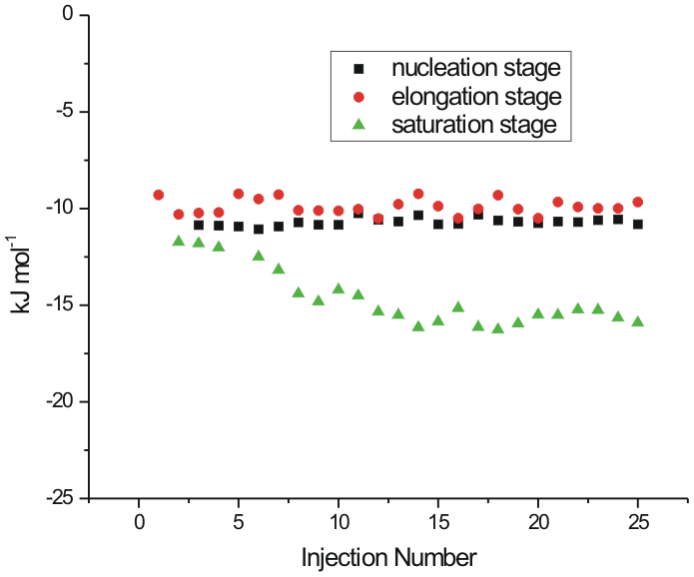
ITC profiles for the titrations of lysozyme solution at different stages with 0.25 M TMAO at 25°C.

Figure 9 represents dilution corrected integrated heat profiles for the titration of fibrils taken after 48 hrs, 61 hrs and 90 hrs of incubation into a solution containing 0.25 M TMAO in the sample cell. It is observed that with 0.25 M TMAO, the protein does not show significant heat of interaction either at the nucleation phase or elongation phase of the fibrillization process. In case of matured fibrils the heat change moves towards exothermic effects as the concentration of the fibrils increases in the solution. Experiments could not be performed at further higher concentration of TMAO due to saturating heat signals.

### Differential Scanning Calorimetry

**Figure 10:**
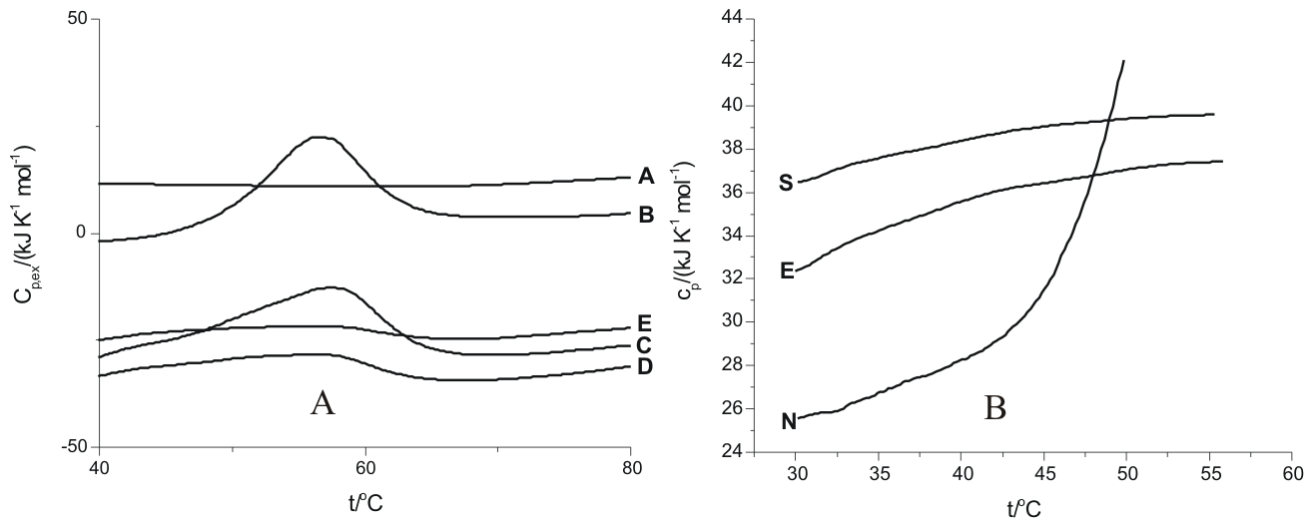
DSC scans of 10 mg ml^−1^ lysozyme (A) at the different stages of fibrillization process: line A represents only buffer, B represents for naïve lysozyme, line C represents for nucleation phase after 48 h of incubation, line D represents for elongation period after 61 h of incubation and line E represents for saturation phase after 90 h of incubation and (B) showing change in heat capacity at the different stages of fibrillization process: line N represents C_p_ for native stage, line E represents C_p_ for elongated stage and line S represents C_p_ for saturation stage.

The differential scanning calorimetry of native lysozyme and at nucleation, elongation and saturation stages of fibrillization was carried out as shown in Figure 10. A solution of 10 mg ml^−1^ lysozyme was incubated at 57°C for different time periods to obtain species at different stages of lysozyme fibrillization process. Curve A shows the DSC scan of buffer verses buffer in the temperature range of 40 to 80°C. Curve B shows the DSC profile of thermal unfolding of native lysozyme in buffer only. The native lysozyme unfolds at (57.4±0.1)°C which is consistent with the transition temperature of the protein reported in literature [9] at pH 2.1. The curve C shows the thermal unfolding of lysozyme at the nucleation stage which corresponds to point II of Figure 1B. The thermal unfolding profile at this stage still shows a significant endothermic transition suggesting presence of significant native like structure in the protein. The DSC scan of lysozyme after 61 h of incubation which corresponds to point III of Figure 1B is shown curve D. This curve belongs to the elongation phase of the fibrillization process. It is seen that the native like properties of lysozyme are largely lost at the elongation stage. Further, after 90 h of incubation corresponding to the saturation phase (Figure 1B), the endotherm is completely lost as seen in curve E of Figure 10A. These results show that as the course of fibrillization proceeds the native like properties of lysozyme are diminished.

Figure 10B shows excess heat capacity over and above the base line for the native state of the protein and at its elongation and saturation stages of fibrillization. It is seen that the excess heat capacity of the protein in the pre-tansition region varies in the following order.
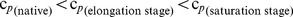
The extent of aggregation is maximum in the saturation state where matured fibrils have been formed compared to that in the elongation state, where the fibril formation is still in progress. Therefore the excess heat capacity of the protein in the saturation state is observed to be highest (Figure 10B).

## Conclusions

The amyloid fibrils of lysozyme have been prepared and monitored by fluorescence spectroscopy using binding properties of ThT. The ThT fluorescence emission intensities upon binding to lysozyme at nucleation, elongation and saturation phases of fibrillization permitted assessment of these stages and planning the ITC experiments to understand the energetics of interaction of the chosen osmolytes with the fibrils. The transmission electron microscopy permitted visualization of these fibrils at nucleation, elongation and fibrillization stages. It was observed that L-proline, 4-hydroxy-L-proline, sarcosine and TMAO prevent fibrillization. It was also observed that 4-hydroxy-L-proline and sarcosine are able to delay the onset of elongation phase by a substantially longer time. However, with TMAO lysozyme solution showed amorphous aggregates even after 300 minutes. The TEM images support the above mentioned observations. The interaction of lysozyme is observed to be exothermic with L-proline and 4-hydroxy-L-proline at the nucleation phase suggesting involvement of polar interactions. These interactions are lesser exothermic at the elongation phase with L-proline and 4-hydroxyl-L-proline compared to that of sarcosine. However, sarcosine undergoes significant polar exothermic interactions with the fibrils at elongation stage. The results suggest that the interaction of the osmolytes with lysozyme fibrils at the nucleation stage involving predominantly polar interactions are major step in the prevention of aggregation/fibrillization, in addition to possibility of inhibition at the elongation phase. In addition to the qualitative information available from the spectroscopic and microscopic measurements, the ITC results have added substantial quantitative understanding on the nature of interactions of these osmolytes with the protein at different stages of the fibrilization process. The DSC results have not only demonstrated the thermal stability of the protein as the fibrilization proceeds, it has also supported the observations on the extent of fibrilization based on the relative changes in the values of heat capacity (Figure 10).

## References

[1] Rousseau F, Schymkowitz J, Serrano L (2006) Protein aggregation and amyloidosis: confusion of the kinds? Curr Opin Struct Biol 16: 118–126 doi: 10.1016/j.sbi.2006.01.011 16434184 10.1016/j.sbi.2006.01.011

[2] Dobson CM (2004) Principles of protein folding, misfolding and aggregation. Semin Cell Dev Biol 15: 3–16 doi: 10.1016/j.semcdb.2003.12.008 15036202 10.1016/j.semcdb.2003.12.008

[3] Dobson CM (2003) Protein folding and disease: a view from the first Horizon Symposium. Nat Rev Drug Discov 2: 154–160 doi: 10.1038/nrd1013 12563307 10.1038/nrd1013

[4] Librizzi F, Rischel C (2005) The kinetic behavior of insulin fibrillation is determined by heterogeneous nucleation pathways. Protein Sci 14: 3129–3134 doi: 10.1110/ps.051692305 16322584 10.1110/ps.051692305 PMC2253244

[5] Kelly JW (1998) The alternative conformations of amyloidogenic proteins and their multi-step assembly pathways. Curr Opin Struct Biol 8: 101–106 doi: 10.1016/S0959-440X(98)80016-X 9519302 10.1016/s0959-440x(98)80016-x

[6] Uversky VN, Fink AL (2004) Conformational Constraints for Amyloid Fibrillation: The Importance of Being Unfolded. Biochim Biophys Acta 1698: 131–153 doi: 10.1016/j.bbapap.2003.12.008 10.1016/j.bbapap.2003.12.008 15134647

[7] Arakawa T, Timasheff SN (1982a) Stabilization of protein structure by sugars. Biochemistry 21: 6536–6544 doi: 10.1021/bi00268a033 7150574 10.1021/bi00268a033

[8] Arakawa T, Timasheff SN (1982b) Preferential interactions of proteins with salts in concentrated solutions. Biochemistry 21: 6545–6552 doi: 10.1021/bi00268a034 7150575 10.1021/bi00268a034

[9] Kar K, Kishore N (2007) Enhancement of thermal stability and inhibition of protein aggregation by osmolytic effect of 4-hydroxy-L-proline. Biopolymers 87: 339–351 doi: 10.1002/bip.20834 17764077 10.1002/bip.20834

[10] Dong XY, Huang Y, Sun Y (2004) Refolding kinetics of denatured-reduced lysozyme in the presence of folding aids. J Biotechnol 114: 135–142 doi: 10.1016/j.jbiotec.2004.06.012 10.1016/j.jbiotec.2004.06.012 15464607

[11] Ahmad FB, Williams PA (1999) Effect of sugars on the thermal and rheological properties of sago starch. Biopolymers 50: 401–412 doi: 10.1002/(SICI)1097-0282(19991005)5 10423549 10.1002/(SICI)1097-0282(19991005)50:4<401::AID-BIP6>3.0.CO;2-V

[12] Mishra R, Seckler R, Bhat R (2005) Efficient refolding of aggregation prone citrate synthase by polyol osmolytes: How well are folding and stability aspects coupled? J Biol Chem 280: 15553–15560 doi: 10.1074/jbc.M410947200 15695514 10.1074/jbc.M410947200

[13] Yoshimoto N, Hashimoto T, Felix MM, Umakoshi H, Kuboi R (2003) Artificial chaperone-assisted refolding of bovine carbonic anhydrase using molecular assemblies of stimuli-responsive polymers. Biomacromolecules 4: 1530–1538 doi: 10.1021/bm015662a 14606877 10.1021/bm015662a

[14] Yang DS, Yip CM, Huang TH, Chakrabartty A, Fraser PE (1999) Manipulating the amyloid-beta aggregation pathway with chemical chaperones. J Biol Chem 274: 32970–32974 doi: 10.1074/jbc.274.46.32970 10551864 10.1074/jbc.274.46.32970

[15] Tanaka M, Machida Y, Nukina N (2005) A novel therapeutic strategy for polyglutamine diseases by stabilizing aggregation-prone proteins with small molecules. J Mol Med 83: 343–352 doi: 10.1007/s00109-004-0632-2 15759103 10.1007/s00109-004-0632-2

[16] Tanaka M, Machida Y, Niu S, Ikeda T, Jana NR, et al (2004) Trehalose alleviates polyglutamine-mediated pathology in a mouse model of Huntington disease. Nat Med 10: 148–154 doi:10.1038/nm985 14730359 10.1038/nm985

[17] Osserman EF, Lawlor DP (1966) Serum and urinary lysozyme (muramidase) in monocytic and monomyelocytic leukemia. J Exp Med 124: 921–952 doi: 10.1084/jem.124.5.921 5224561 10.1084/jem.124.5.921 PMC2138258

[18] Dobson CM, Evans PA, Radford SE (1994) Understanding how proteins fold: the lysozyme story so far. Trends Biochem Sci 19: 31–37 doi: 10.1016/0968-0004(94)90172-4 8140619 10.1016/0968-0004(94)90171-6

[19] Boyer PM, Hsu JT (1992) Effect of ligand concentration on protein adsorption in dye ligand adsorbents. Chem Eng Sci 47: 241–251 doi: 10.1016/0009-2509(92)80218-2

[20] Wall J, Murphy CL, Solomon A (1999) In vitro immunoglobulin light chain fibrillogenesis. Methods Enzymol 309: 204–217 doi: 10.1016/S0076-6879(99)09016-3 10507026 10.1016/s0076-6879(99)09016-3

[21] Kelenyi G (1967) On the histochemistry of azo group-free thiazole dyes. J Histochem Cytochem 15: 172–180 doi: 10.1177/15.3.172 4166508 10.1177/15.3.172

[22] Hobbs JR, Morgan AD (1963) Fluorescence microscopy with thioflavine-T in the diagnosis of amyloid. J Pathol Bacteriol 86: 437–442 DOI: 10.1002/path.1700860218 14068952 10.1002/path.1700860218

[23] Saeed SM, Fine G (1967) Thioflavin T for amyloid detection. Am J Clin Pathol 47: 588–593. 4164576 10.1093/ajcp/47.5.588

[24] Vassar PS, Culling CFA (1959) Fluorescent stains with special reference to amyloid and connective tissues. Arch Pathol 68: 487–494. 13841452

[25] Khurana R, Coleman C, Ionescu-Zanetti C, Carter SA, Krishna V, et al (2005) Mechanism of thioflavin-T binding to amyloid fibrils: localisation and implications. J Struct Biol 151: 229–238 doi: 10.1016/j.jsb.2005.06.006 16125973 10.1016/j.jsb.2005.06.006

[26] Ohi M, Li Y, Cheng Y, Walz T (2004) Negative Staining and Image Classification – Powerful Tools in Modern Electron Microscopy. Biological Procedures 6: 23–34 doi: 10.1251/bpo70 10.1251/bpo70 PMC389902 15103397

[27] LeVine H III (1997) Stopped flow kinetics reveal multiple phases of Thioflavin T binding to Alzhimer β(1–40) amyloid fibrils. Arch Biochem Biophys 342: 306–316 doi: org/10.1006/abbi.1997.0137 9186492 10.1006/abbi.1997.0137

[28] Groenning M, Norrman M, Flink JM, van de Weert M, Bukrinsky JT, et al (2007) Binding mode of ThT in insulin amyloid fibrils. J Struct Biol 159: 483–497 doi: 10.1016/j.jsb.2007.06.004 17681791 10.1016/j.jsb.2007.06.004

[29] Jain S, Udgaonkar JB (2010) Salt-induced modulation of the pathway of amyloid fibril formation by the mouse prion protein. Biochemistry 49: 7615–7624 doi: 10.1021/bi100745j 20712298 10.1021/bi100745j

[30] Munishkina LA, Henriques J, Uversky VN, Fink AL (2004) Role of protein-water interactions and electrostatics in R-synuclein fibril formation. Biochemistry 43: 3289–3300 doi: 10.1021/bi034938r 15023080 10.1021/bi034938r

[31] Yeh V, Broering JM, Romanyuk A, Chen B, Chernoff YO, et al (2010) The Hofmeister effect on amyloid formation using yeast prion protein. Protein Sci 19: 47–56 doi: 10.1002/pro.281 19890987 10.1002/pro.281 PMC2817838

[32] Muzaffar M, Ahmad A (2011) The Mechanism of Enhanced Insulin Amyloid Fibril Formation by NaCl Is Better Explained by a Conformational Change Model. PLoS ONE 6: e27906 doi: 10.1371 22132167 10.1371/journal.pone.0027906 PMC3221682

[33] Kraulis PJ (1991) MOLSCRIPT: a program to produce both detailed and schematic plots of protein structures. J Appl Crystallogr 24: 946–950 doi: 10.1107/S0021889891004399

[34] Ivanova MI, Sawaya MR, Gingery M, Attinger A, Eisenberg D (2004) An amyloid-forming segment of beta2-microglobulin suggests a molecular model for the fibril. Proc Nat Acad Sci USA 101: 10584–10589 doi: 10.1073/pnas.0403756101 15249659 10.1073/pnas.0403756101 PMC489978

[35] Qi W, Zhang A, Good TA, Fernandez EJ (2009) Two disaccharides and trimethylamine N-oxide affect Aβ aggregation differently, but all attenuate oligomer-induced membrane permeability. Biochemistry 48: 8908–8919 doi: 10.1021/bi9006397 19637920 10.1021/bi9006397 PMC2838166

[36] Yang DS, Yip CM, Huang THJ, Chakrabartty A, Fraser PE (1999) Manipulating the amyloid-β aggregation pathway with chemical chaperones. J Biol Chem 274: 32970–32974 doi: 10.1074/jbc.274.46.32970 10551864 10.1074/jbc.274.46.32970

[37] Uversky VN, Li J, Fink AL (2001) Trimethylamine-N-oxide-induced folding of α-synuclein. FEBS Lett 509: 31–35 doi: 10.1016/S0014-5793(01)03121-0 11734201 10.1016/s0014-5793(01)03121-0

[38] Borwankar T, Rothlein C, Zhang G, Techen A, Dosche C, et al (2011) Natural osmolytes remodel the aggregation pathway of mutant huntingtin exon 1. Biochemistry 50: 2048–2060 doi: 10.1021/bi1018368 21332223 10.1021/bi1018368

[39] Auton M, Bolen DW (2005) Predicting the energetics of osmolyte-induced protein folding/unfolding. Proc Natl Acad Sci USA 102: 15065–15068 doi: 10.1073/pnas.0507053102 16214887 10.1073/pnas.0507053102 PMC1257718

[40] Guo F, Friedman JM (2009) Osmolyte-induced perturbations of hydrogen bonding between hydration layer waters: correlation with protein conformational changes. J Phys Chem B 113: 16632–16642 doi: 10.1021/jp9072284 19961206 10.1021/jp9072284 PMC3354986

[41] Kumar TKS, Samuel D, Jayaraman G, Srimathi T, Yu C (1998) The role of L-proline in the prevention of aggregation during protein folding in vitro. . Biochem Mol Biol Int 46: 509–517 doi: 10.1080/15216549800204032 9818090 10.1080/15216549800204032

[42] Ignatova Z, Gierasch LM (2006) Inhibition of protein aggregation in vitro and in vivo by a natural osmoprotectant. Proc Nat Acad Sci USA 36: 13357–13361 doi: 10.1073/pnas.0603772103 10.1073/pnas.0603772103 PMC1569168 16899544

